# Comparative Analysis of CNN and Transformer Models for Multi-Class Diabetic Retinopathy Grading Using Fundus Images

**DOI:** 10.3390/diagnostics16172882

**Published:** 2026-09-07

**Authors:** Maha A. Thafar

**Affiliations:** Department of Computer Science, College of Computers and Information Technology, Taif University, P.O. Box 11099, Taif 21944, Saudi Arabia; m.thafar@tu.edu.sa

**Keywords:** diabetic retinopathy, fundus imaging, deep learning, convolutional neural networks, transformer-based models, transfer learning, multi-class classification

## Abstract

**Background/Objectives:** Diabetic retinopathy is a major cause of preventable vision loss worldwide, making early and accurate disease grading crucial for timely treatment. Although convolutional neural network (CNN)- and transformer-based architectures have demonstrated promising performance for retinal image analysis, comprehensive comparisons within a unified experimental framework remain limited. This study systematically compares representative standard and lightweight CNN- and transformer-based architectures for multi-class DR grading. **Methods:** Six ImageNet-pretrained deep-learning models, including ResNet50, EfficientNet-B0, MobileNetV2, Vision Transformer (ViT), Swin-Tiny, and Swin Transformer, were evaluated on the APTOS 2019 retinal fundus image dataset under a unified experimental configuration with consistent preprocessing, data augmentation, training, and evaluation settings. All models were fine-tuned and evaluated independently over five runs with different random seeds. Their performance was assessed using accuracy, precision, recall, F1-score, area under the receiver operating characteristic curve (AUC), Quadratic Weighted Kappa (QWK), per-class analysis, computational efficiency, and statistical analysis. **Results:** Transformer-based models generally achieved higher mean classification performance than the evaluated CNN-based models. Swin-Tiny achieved the highest mean accuracy (82.3%), macro F1-score (64.4%), weighted F1-score (82.1%), and QWK (89.8%) across the five runs. Among the CNN-based models, EfficientNet-B0 achieved the strongest overall classification performance, whereas MobileNetV2 provided the lowest computational complexity. The results also highlighted differences in learning behavior and computational requirements across the evaluated architectures. Repeated experiments demonstrated stable performance across different random seeds, supporting the reliability of the proposed evaluation. **Conclusions:** Overall, this study provides a comprehensive comparison of representative CNN- and transformer-based architectures under consistent experimental settings and offers practical guidance for selecting suitable deep learning models for automated diabetic retinopathy screening.

## 1. Introduction

Retinal diseases are responsible for a substantial proportion of cases of vision impairment and blindness worldwide, posing an increasing challenge to healthcare systems [1]. These disorders, including diabetic retinopathy (DR), pathological myopia (PM), age-related macular degeneration (AMD), and glaucoma (GLA), impose a substantial burden on healthcare systems and significantly affect quality of life, particularly among aging and working-age populations [2]. Early detection and regular monitoring of retinal abnormalities play a crucial role in preventing permanent vision loss.

Among these conditions, diabetic retinopathy (DR), a microvascular complication of diabetes, is one of the most prevalent and clinically significant causes of blindness [2,3]. The global burden of DR continues to increase, with the number of affected individuals projected to rise from approximately 126.6 million in 2010 to 191.0 million by 2030. In particular, cases of vision-threatening diabetic retinopathy (VTDR) are expected to grow from 37.3 million to 56.3 million if timely intervention is not achieved [4]. Despite available effective screening programs and treatment strategies, DR remains a leading cause of vision loss among working-age populations [5]. Early detection and accurate grading of disease severity are essential for timely intervention and effective treatment planning. However, manual examination of retinal fundus images is time-consuming, requires expert ophthalmologists, and is subject to inter-observer variability.

The rapid evolution of artificial intelligence (AI) has accelerated the adoption of deep learning (DL) for automated medical image analysis. This technology has significantly improved the accuracy of disease detection across various image modalities [5,6,7,8], including various retinal diseases [9,10]. Transfer learning (TL) and pretrained models have further improved performance in data-limited clinical settings [11]. In particular, convolutional neural networks (CNNs) perform strongly in retinal disease classification tasks due to their ability to learn hierarchical spatial representations from fundus images [12]. Despite their success, CNN-based models primarily focus on local feature extraction and may struggle to capture global contextual relationships, which can be critical for distinguishing subtle differences in diabetic retinopathy severity levels. More recently, transformer-based models have emerged as an alternative paradigm in computer vision, offering the capability to model global contextual relationships through self-attention mechanisms [13]. These models have shown competitive performance in various image classification tasks.

While both CNN-based and transformer-based models have demonstrated promising performance, their relative effectiveness for fine-grained multi-class classification tasks, such as diabetic retinopathy grading, remains an active research topic. As deep learning architectures continue to evolve, consistent comparative evaluations conducted under identical experimental settings are needed to test their relative strengths, limitations, and practical applicability in automated retinal screening systems. Moreover, few studies have investigated the trade-off between classification performance and computational efficiency, an important consideration for deploying deep learning models in practical retinal screening systems.

To address these gaps, this study presents a comprehensive comparative analysis of six state-of-the-art pretrained deep learning architectures for five-class diabetic retinopathy grades using retinal fundus images. Three representative CNN-based models, namely ResNet50, EfficientNet-B0, and MobileNetV2, are compared with three transformer-based models, including Vision Transformer (ViT), Swin-Tiny Transformer, and Swin Transformer. These models are trained and evaluated under identical preprocessing, augmentation, transfer learning, training protocols, and evaluation criteria to ensure a fair comparison and to provide a consistent experimental protocol for comparative evaluation. In addition to prediction performance, this study analyzes each architecture’s computational efficiency in terms of model complexity and inference speed, providing practical insights for selecting suitable DL models for automated diabetic retinopathy screening.

The key contributions of this study are summarized as follows:

• A unified experimental framework for the comparative evaluation of representative CNN-based and transformer-based architectures for five-class diabetic retinopathy grading.
• A systematic evaluation of six pretrained deep learning models, including ResNet50, EfficientNet-B0, MobileNetV2, Vision Transformer, Swin-Tiny Transformer, and Swin Transformer, using transfer learning.
• Investigation of multi-class diabetic retinopathy grading under an imbalanced dataset using a unified training strategy based on weighted loss and balanced sampling.
• A comprehensive comparative analysis consisting of overall and per-class classification performance, QWK, learning and convergence behavior, computational efficiency, and performance stability across five independent runs.

## 2. Related Work

Deep learning has become the dominant approach for automated diabetic retinopathy grading, leading to the development of numerous CNN-based and transformer-based architectures. Existing studies have explored various strategies to improve classification performance, ranging from the design of more effective convolutional backbones to the adoption of self-attention mechanisms for global feature modeling [14,15,16]. This section reviews recent research in three categories: CNN-based approaches, transformer-based approaches, and comparative studies that motivate the present study.

### 2.1. CNN-Based Approaches for Diabetic Retinopathy Classification

CNN-based methods remain the foundation for automated DR grading, largely due to their strong performance on fundus imagery and their compatibility with transfer learning. Early work focused on adapting established backbones such as VGG and ResNet, then progressively shifted toward more parameter-efficient and task-specific designs, including EfficientNet and its variants through transfer learning.

One line of research refines classical VGG-style networks [17]. The VGG-NiN architecture augments VGG16 with spatial pyramid pooling and network-in-network blocks to better capture scale-varying lesions while reducing parameters, enabling robust multiclass grading at varying image resolutions compared with vanilla VGG and traditional pipelines [17]. Building on the advantages of pretraining, Saeed et al. introduce an adaptive fine-tuned CNN that reinitializes and selectively fine-tunes early convolutional layers on DR data, replaces fully connected layers with a PCA-based feature stage, and employs gradient-boosted classifiers, which together improve generalization on EyePACS and Messidor for multiclass diagnosis [18].

As CNN architectures continued to mature, research shifted toward designing more specialized networks for diabetic retinopathy analysis. A pretrained NASNet-based framework, referred to as DR-NASNet, incorporated Ben Graham preprocessing, CLAHE-based contrast enhancement, data augmentation, and dense blocks to improve feature extraction while maintaining a relatively compact model with lower computational complexity [19].

Similarly, a CNN with residual blocks (DRCNNRB) combined image preprocessing with geometric data augmentation, including rotation, flipping, shearing, zooming, and rescaling, to alleviate class imbalance and improve classification robustness. The proposed framework demonstrated superior diagnostic performance and computational efficiency compared with several existing CNN-based approaches [20]. More recently, lightweight CNN architectures have received increasing attention for practical DR screening. An enhanced MobileNet framework introduced architectural modifications, including Swish activation functions, depthwise separable convolutions, and improved dropout regularization, achieving competitive classification performance while outperforming several benchmark models, including ResNet101, DenseNet121, EfficientNet-B7, and hybrid CNN-based approaches, with substantially lower computational complexity. These findings highlight the potential of lightweight CNN models for resource-constrained and edge-based healthcare applications [21].

As the availability of retinal image datasets increased, researchers began systematically comparing different CNN architectures for diabetic retinopathy grading. One study systematically evaluated VGG, ResNet, and EfficientNet variants [22], and showed that EfficientNet-B3 yields superior performance for five-class DR grading, reflected in a quadratic weighted kappa of 0.85 that better accounts for ordinal misclassification than raw accuracy. Similarly, another study [23] investigated the performance of multiple CNN architectures, including VGG16, ResNet50, and ResNet101, for multiclass DR classification using a large clinical fundus image dataset. Their framework incorporated image preprocessing, data augmentation, and regularization to address data imbalance and improve model generalization. Among the evaluated models, ResNet101 consistently outperformed ResNet50 and VGG16, demonstrating the effectiveness of deeper residual networks for retinal image classification. Their findings further demonstrated the advantage of residual learning over conventional CNN architectures, motivating the inclusion of ResNet-based models in subsequent comparative studies.

Beyond improving standalone CNN architectures, recent studies have explored hybrid deep learning frameworks that integrate CNNs with complementary learning techniques to further enhance diabetic retinopathy classification. One such framework combined retinal image preprocessing, lesion segmentation, transfer learning using SqueezeNet, feature dimensionality reduction, and a He-Weighted Bi-directional Long Short-Term Memory (HWBLSTM) classifier to exploit both spatial and sequential feature representations. The proposed approach achieved competitive performance on the APTOS and MESSIDOR datasets while maintaining relatively low computational overhead, demonstrating the potential of combining CNN-based feature extraction with advanced learning modules for DR grading [24].

Overall, CNN-based approaches have evolved from conventional transfer learning using standard architectures toward specialized network designs, lightweight architectures, comparative evaluations of modern backbones, and hybrid learning frameworks. Despite these advances, most CNN models rely primarily on convolutional operations to capture local spatial information, which may limit their ability to model long-range contextual relationships in retinal fundus images. This limitation has motivated the growing adoption of transformer-based architectures that employ self-attention mechanisms to jointly capture local and global image representations.

### 2.2. Transformer-Based Approaches for Retinal Image Analysis

Transformer-based architectures have recently emerged as a promising alternative to convolutional neural networks for retinal image analysis, particularly for diabetic retinopathy classification [13,25]. Unlike CNNs, which mainly learn local spatial representations through convolutional operations, transformers employ self-attention to model relationships between distant image regions and learn global contextual representations. These characteristics make transformer models particularly suitable for diabetic retinopathy grading, where disease severity is often determined by multiple lesions distributed throughout the retinal fundus.

Early studies demonstrated the feasibility of applying pure Vision Transformers (ViTs) to diabetic retinopathy classification. A patch-based ViT framework showed that transformer models could achieve competitive performance for five-class DR grading while outperforming several conventional CNN-based approaches, highlighting the effectiveness of global attention mechanisms for retinal image analysis [26]. As transformer models continued to evolve, research increasingly focused on overcoming their dependence on large, annotated datasets and improving feature representation. Self-supervised learning strategies were introduced to pretrain Vision Transformers using unlabeled retinal images before fine-tuning for diabetic retinopathy grading, substantially improving label efficiency while maintaining competitive classification performance [27]. Similarly, optimized Vision Transformer frameworks specifically designed for retinal disease analysis, called OcuViT, show that carefully fine-tuned ViT-Base models, combined with streamlined preprocessing, can achieve state-of-the-art performance on both binary and five-class DR grading (APTOS) as well as AMD grading, outperforming earlier CNN- and ViT-based systems in accuracy and robustness [28].

Recent studies have also explored architectural improvements to better exploit the high resolution and heterogeneity of retinal fundus images. Transformer with Multiple Instance Learning (TMIL) introduced high-resolution fundus images segmentation into 224 × 224 patches. After that, a patch aggregation strategy was applied using a dedicated Global Instance Computing Block. This design allows reuse of pre-trained ViT weights without image down-scaling, improving accuracy, minimizing the computational cost, and reducing inference time by 62% compared with naïvely feeding high-resolution images into ViT, enabling more efficient analysis of retinal images without excessive image down-sampling [29].

More recently, research has focused on developing advanced transformer architectures that improve robustness, computational efficiency, and feature representation for retinal image analysis. Swin-DRNet [30] introduced a hierarchical Swin Transformer framework that combines contrast-adaptive preprocessing, class-aware representation learning, and focal-loss optimization to improve diabetic retinopathy screening under heterogeneous imaging conditions and severe class imbalance. Experimental evaluation on the APTOS 2019, IDRiD, and Messidor2 datasets demonstrated consistent performance across different datasets, highlighting the strong generalization capability of hierarchical transformers for real-world DR screening. Similarly, an Attention Dual Transformer with Adaptive Temporal Convolution (ADT-ATC) [31] incorporated dual spatial transformer modules, adaptive temporal convolution, and hierarchical cross-attention to model fine retinal lesions and global contextual information jointly. The proposed framework achieved superior classification performance compared with conventional deep learning approaches, demonstrating the potential of advanced attention mechanisms for diabetic retinopathy detection.

While pure ViTs and their variants improve global context modeling, several studies argue that the most effective DR grading systems combine transformers with CNNs to exploit both local lesion sensitivity and long-range relational reasoning. Hybrid models thus emerge as a natural next step after CNN and pure ViT pipelines. Therefore, recent research has investigated hybrid CNN–Transformer frameworks that combine the complementary strengths of convolutional feature extraction and self-attention mechanisms. Representative approaches integrate residual or densely connected CNN backbones with transformer modules to simultaneously capture local retinal lesions and long-range contextual relationships, leading to improved multiclass DR grading performance compared with standalone CNN or transformer models. Examples of these frameworks include: CTNet (combines residual CNN with transformer) [32], and D-TNet (couples DenseNet121 with a Transformer architecture) [33].

Overall, transformer-based approaches have progressed from conventional vision transformers toward improved optimization strategies, self-supervised learning, hierarchical attention mechanisms, and hybrid CNN–Transformer frameworks. These developments have substantially advanced automated diabetic retinopathy grading and demonstrated the effectiveness of attention-based models for retinal image analysis.

### 2.3. Research Gap and Motivation

The reviewed literature demonstrates substantial progress in automated diabetic retinopathy grading. CNN-based approaches have evolved from conventional transfer learning frameworks toward specialized, lightweight architectures, while transformer-based methods have introduced self-attention mechanisms, hierarchical feature learning, and hybrid CNN–transformer frameworks to further improve classification performance. These advances have significantly enhanced deep learning’s capabilities for retinal image analysis.

Despite these developments, some studies have compared CNN- and transformer-based models for diabetic retinopathy classification. However, differences in the selected architectures, preprocessing pipelines, transfer learning strategies, training protocols, and evaluation criteria make direct comparison across studies challenging. Moreover, most existing studies primarily emphasize predictive performance, with limited comprehensive evaluation of classification performance, class-specific behavior, performance stability, learning behavior, and computational efficiency within a common experimental framework. Therefore, a consistent and controlled benchmark using a representative standard and lightweight CNN and transformer architectures remains valuable for understanding the trade-offs between predictive performance and computational cost. Motivated by those gaps, this study presents a unified comparative evaluation of representative pretrained CNN and transformer architectures for five-class diabetic retinopathy grading using consistent preprocessing, training, and evaluation settings.

## 3. Materials and Methods

### 3.1. The Proposed Framework Overview

**Figure 1:**
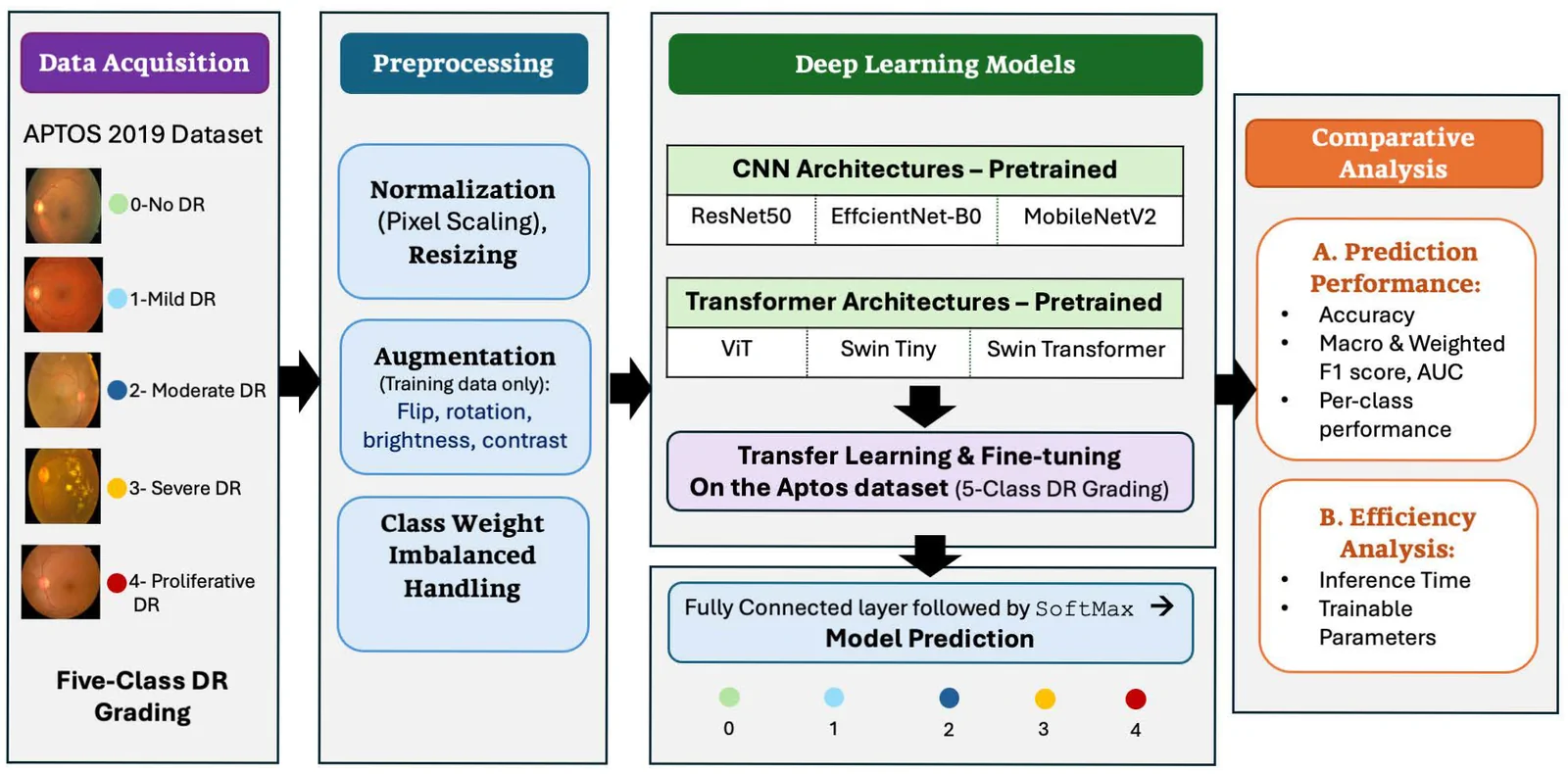
Overall workflow of the proposed framework for multi-class diabetic retinopathy grading.

In this study, diabetic retinopathy (DR) grading is formulated as a supervised multi-class classification task. The objective is to automatically classify retinal fundus images into five DR severity levels, ranging from no apparent DR to advanced disease stages. The proposed methodological framework, illustrated in Figure 1, consists of seven main steps:

1. **Data Acquisition:** Retinal fundus images representing the five DR severity classes are collected from publicly available datasets.
2. **Data Splitting** into training, validation, and testing subsets for model development and evaluation.
3. **Data Preprocessing:** Standardization of input images to ensure compatibility across different DL architectures.
4. **Data Augmentation and Class Imbalance Handling:** Apply data augmentation and training strategies to improve model generalization and address class distribution imbalance.
5. **Transfer Learning using Pretrained DL Models:** Adaptation of CNN- and transformer-based pretrained models for multi-class DR grading.
6. **Classification:** Prediction of DR severity grades using the learned representations and classification layers.
7. **Model Evaluation and Analysis:** Evaluation and comparison of model performance by the use of standard evaluation metrics and computational analysis.

The following subsections describe each stage of the proposed workflow in detail.

### 3.2. Dataset Acquisition and Statistics

In this study, the APTOS 2019 blindness detection dataset was utilized for multi-class diabetic retinopathy (DR) grading [34]. The dataset is publicly available through the Kaggle repository and was originally released as part of the Asia Pacific Tele-Ophthalmology Society (APTOS) challenge. It consists of 3662 RGB retinal fundus images that were clinically examined and annotated by trained ophthalmologists according to the International Clinical Diabetic Retinopathy Disease Severity Scale (ICDRSS) [35]. Each fundus image is assigned to one of five DR severity grades: no diabetic retinopathy (No DR), mild DR, moderate DR, severe DR, and proliferative DR.

The publicly available dataset includes predefined training, validation, and testing subsets, which were directly adopted in this study without performing any additional data splitting to ensure reproducibility. The predefined subsets preserve a similar class distribution across the training (approximately 80%), validation (10%), and test (10%) sets, resulting in an approximately stratified distribution across the five DR severity grades, as summarized in Table 1. The dataset download source is provided in the Data Availability Statement. However, patient-level information is not provided with the original APTOS 2019 data, and detailed information on how the predefined subsets were generated is not available from the repository. Therefore, patient-level independence across the training, validation, and test subsets could not be verified. As shown in Table 1, the dataset exhibits a clear class imbalance, with a substantially larger number of normal retinal images than advanced DR stages. This characteristic was considered during model training by employing class-balanced optimization strategies, as described in Section 4.

**Figure 2:**
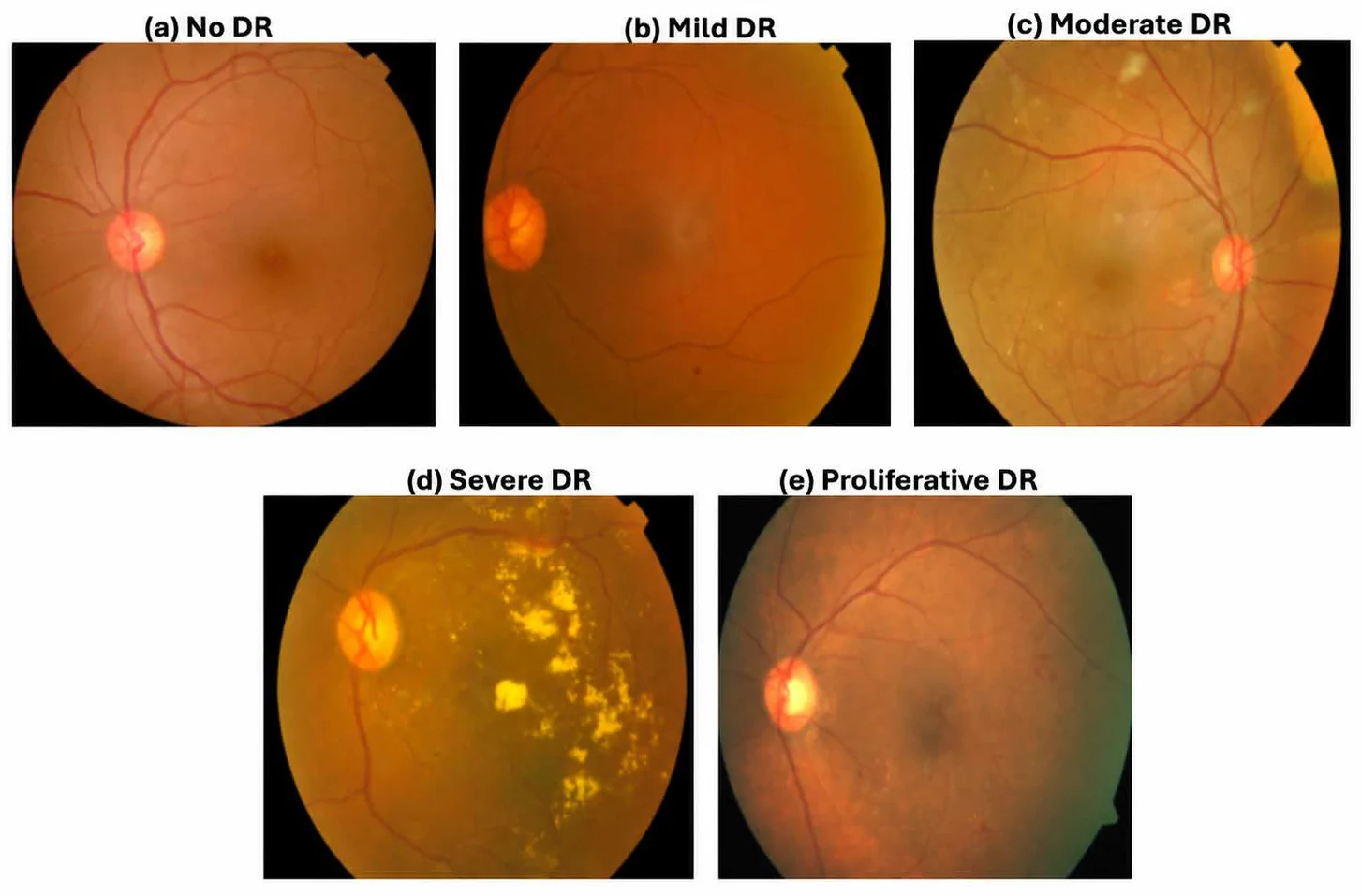
Representative retinal fundus images illustrating the five diabetic retinopathy (DR) severity grades from the APTOS 2019 dataset.

Representative retinal fundus images corresponding to the five diabetic retinopathy severity grades are presented in Figure 2. These examples illustrate the progressive visual characteristics of diabetic retinopathy, ranging from normal retinal appearance to proliferative disease.

### 3.3. Data Preprocessing

All retinal fundus images were processed using a unified preprocessing pipeline to ensure consistency across DL architectures and enable fair comparison between CNN- and transformer-based models. First, all images were converted into RGB format to maintain a consistent three-channel representation. The images were then resized to a fixed resolution of 224 × 224 pixels to meet the input requirements of the selected pretrained models.

Pixel intensity values were normalized to provide a standardized input distribution and improve training stability. Since the models were initialized with weights pretrained on ImageNet, the images were normalized using the corresponding ImageNet mean and standard deviation. Additionally, the categorical diabetic retinopathy severity labels were converted to numerical class labels corresponding to the five grading levels: No DR (0), Mild DR (1), Moderate DR (2), Severe DR (3), and Proliferative DR (4). The same preprocessing procedure was applied across all evaluated architectures to ensure that performance differences were primarily attributable to model characteristics rather than variations in data preparation.

### 3.4. Data Augmentation and Class Imbalance Handling

To improve model generalization and reduce overfitting, data augmentation was applied only to the training set. The augmentation pipeline consisted of random horizontal flipping, small-angle rotation, slight geometric transformations, and mild brightness and contrast adjustments. No augmentation was applied to the validation or test images to ensure unbiased model evaluation. To reduce the effect of class imbalance among DR severity grades, a class-weighted loss function was employed during training, assigning higher penalties to underrepresented classes.

### 3.5. Selection of Pretrained Deep Learning Architectures

To investigate the performance differences between convolution-based and attention-based architectures, this study selected representative pretrained models from CNN and transformer families. The selected CNN models (ResNet50, EfficientNet-B0, and MobileNetV2) represent complementary convolutional design strategies, namely residual learning, compound model scaling, and lightweight network design. Similarly, the selected transformer models (ViT, Swin-Tiny Transformer, and Swin Transformer) represent complementary attention-based paradigms, including global self-attention and hierarchical window-based attention, while covering both lightweight and higher-capacity architectures. This selection enables a comprehensive comparison of classification performance and computational requirements for multi-class diabetic retinopathy grading.

#### 3.5.1. CNN-Based Models

To evaluate the effectiveness of convolution-based architectures for diabetic retinopathy grading, three representative CNN models were selected: ResNet50, EfficientNet-B0, and MobileNetV2. These models represent different CNN design strategies, including deep residual learning, efficient model scaling, and lightweight architectures. CNN-based models are particularly effective at extracting local spatial patterns from retinal images, which are important for identifying DR-related abnormalities, including lesions, texture variations, and structural changes.

**ResNet50:**

ResNet50 [36] is a deep CNN architecture based on residual learning, in which shortcut connections are introduced to improve gradient flow and mitigate the degradation problem associated with training deeper networks. Through multiple convolutional layers, ResNet50 learns hierarchical visual representations ranging from low-level features, such as edges and textures, to more complex disease-related patterns. In this study, ResNet50 was selected as a standard CNN baseline to evaluate the capability of deep residual architectures for multi-class DR grading.

**EfficientNet-B0:**

EfficientNet-B0 [37] is based on compound scaling, which systematically balances network depth, width, and input resolution to improve performance while maintaining computational efficiency. This balanced scaling allows the model to learn representative visual features with fewer parameters compared with conventional CNN architectures. EfficientNet-B0 was included to investigate the relationship between classification performance and computational efficiency in retinal image analysis.

**MobileNetV2:**

MobileNetV2 [38] is a lightweight CNN architecture designed to reduce computational complexity through depth wise separable convolutions and inverted residual blocks. These mechanisms reduce the number of trainable parameters while preserving effective feature extraction. In this study, MobileNetV2 was evaluated to assess the potential of lightweight CNN architectures for efficient DR grading, particularly in scenarios with limited computational resources.

#### 3.5.2. Transformer-Based Models

To compare convolution-based models with attention-based approaches, three transformer architectures were evaluated: Vision Transformer (ViT), Swin-Tiny Transformer, and Swin Transformer. Unlike CNNs, which primarily focus on local feature extraction, transformer-based models use self-attention mechanisms to capture relationships between different image regions. This capability may be beneficial for retinal image analysis, where disease severity can depend on abnormalities distributed across different areas of the fundus image.

**Vision Transformer (ViT):**

ViT [39] adapts transformer architectures for image classification by dividing an image into fixed-size patches and modeling relationships among these patches using self-attention mechanisms. This design enables the model to capture global contextual information across the entire retinal image. ViT was selected as the standard transformer baseline to evaluate the effectiveness of global attention-based representation learning for DR grading.

**Swin-Tiny Transformer:**

Swin-Tiny Transformer [40] is a lightweight hierarchical transformer architecture that introduces shifted window-based self-attention. By limiting attention computation within local windows while enabling information exchange between regions, it reduces computational complexity while maintaining contextual feature learning. Swin-Tiny was included to investigate the performance of efficient transformer architectures compared with lightweight CNN models.

**Swin Transformer (Base variant):**

Swin Transformer [40] extends the vision transformer concept by incorporating hierarchical feature extraction and shifted-window attention mechanisms, enabling the model to capture multi-scale representations. This architecture combines local feature modeling with broader contextual understanding, which can be valuable for analyzing retinal abnormalities appearing at different scales. In this study, Swin Transformer was selected as a stronger hierarchical transformer architecture for comparison with CNN-based models.

The selected architectures allow analysis of model behavior across different design families, including CNN versus transformer models, standard versus lightweight architectures, and accuracy–efficiency trade-offs.

### 3.6. Transfer Learning, Fine-Tuning, and Classification Strategy

To leverage knowledge learned from large-scale pretrained architectures, this study adopted a transfer learning approach for multi-class diabetic retinopathy grading from retinal fundus images. As described in Section 3.4, the selected CNN-based and transformer-based models were pretrained on large-scale image datasets and have demonstrated strong capabilities in extracting meaningful visual representations. Through transfer learning, these pretrained representations were adapted to the DR classification task, reducing the need for training models from scratch and improving the learning process when using a limited amount of medical imaging data.

To adapt each pretrained architecture for the proposed classification task, the original classification layer was replaced with a task-specific classification head consisting of five output neurons corresponding to the five DR severity grades: Class 0: No DR; Class 1: Mild DR; Class 2: Moderate DR; Class 3: Severe DR; and Class 4: Proliferative DR. The output of each model was passed through a SoftMax activation function to generate a probability distribution across the five classes, where the class with the highest probability score was selected as the final predicted DR grade.

During fine-tuning, all pretrained model parameters, including the backbone and the task-specific classification head, were optimized using the training data to adapt the learned feature representations to retinal fundus images. The validation set was used to monitor model performance and select the best-performing model checkpoint during training. The same fine-tuning strategy was applied across all evaluated architectures to provide a consistent experimental setting for comparing CNN-based and transformer-based models.

## 4. Experimental Setup and Evaluation Protocols

### 4.1. Implementation Environment

The proposed framework was implemented in Python 3.13.15 using the PyTorch 2.11.0 deep learning framework. Torchvision 0.26.0 was employed for image preprocessing and data augmentation, while pretrained CNN and transformer models were loaded using the TIMM library. The TIMM 1.0.28 model identifiers used in our experiments were resnet50, efficientnet_b0, mobilenetv2_100, vit_base_patch16_224, swin_tiny_patch4_window7_224, and swin_base_patch4_window7_224. Model evaluation and visualization were performed using Scikit-learn 1.6.1 and Matplotlib 3.10.0, respectively. Additional libraries, including Pillow and TQDM, were used for image handling and monitoring the training process. All experiments were conducted using Google Colab Pro+ with a Python 3 runtime and NVIDIA A100 GPU acceleration. To ensure consistency throughout the experiments, all models were trained and evaluated under the same hardware environment and software configuration. Furthermore, five independent experiments were performed using different random seeds, while the random seeds for Python, NumPy 2.1.3, and PyTorch were fixed within each run to ensure reproducibility.

### 4.2. Hyperparameter Configuration and Training Setup

All models were trained using the same experimental configuration to ensure a consistent comparison between the evaluated architectures. The models were initialized with ImageNet pretrained weights and fully fine-tuned using the training set, while the validation set was used to monitor model performance, guide early stopping, and select the best-performing checkpoint during training. Several hyperparameters were considered during model optimization, including the learning rate, batch size, optimizer, and number of training epochs. The AdamW optimizer was employed with an initial learning rate of 1 × 10^−4^, a weight decay of 1 × 10^−4^, and a batch size of 32 for all evaluated models. All models were trained for a maximum of 20 epochs. Early stopping with a patience of six epochs was employed based on the validation loss to reduce overfitting and prevent unnecessary training. The selected training configuration was then applied consistently across all evaluated architectures during model training and evaluation. To address the variability introduced by random initialization and stochastic training, each model was independently trained and evaluated five times using different random seeds. The same training configuration and data splitting were maintained across all runs to ensure a fair comparison. Classification results were aggregated across the five runs and reported as mean ± standard deviation (std), providing a more reliable assessment of model performance and stability than a single experimental run. The resulting run-level performance was also used for the statistical analysis of differences among the evaluated models.

To mitigate class imbalance, Weighted Cross-Entropy Loss together with a WeightedRandomSampler was adopted during training. The final hyperparameter configuration used in this study is summarized in Table 2.

### 4.3. Evaluation Metrics

To comprehensively evaluate the performance of the selected CNN-based and transformer-based models for multi-class diabetic retinopathy grading, two categories of evaluation criteria were considered: classification performance and computational efficiency.

For classification performance, all models were evaluated using accuracy, precision, recall, F1-score, and the area under the receiver operating characteristic curve (AUC-ROC). Since the APTOS dataset exhibits an imbalanced distribution across the five DR severity classes, both macro-average and weighted-average precision, recall, F1-score, and AUC were reported. For multiclass AUC, a one-vs.-rest (OvR) strategy was employed, where each DR severity label was evaluated against the remaining labels. The resulting class-specific AUC values were then summarized using macro-average and weighted-average. Macro-average metrics assign equal importance to each class regardless of its size, providing a better assessment of the model’s performance on minority classes. In contrast, weighted-average metrics account for the number of samples in each class, providing an overall performance measure that reflects the class distribution. These complementary metrics offer a more comprehensive evaluation of model performance under class imbalance.

Since diabetic retinopathy grading is an ordinal classification problem, Quadratic Weighted Kappa (QWK) [41] was additionally employed to quantify the agreement between the predicted and true DR severity grades while accounting for the severity of misclassification. In addition, QWK is less affected by class imbalance than conventional classification metrics, making it particularly suitable for evaluating the imbalanced APTOS2019 dataset. Unlike standard classification metrics, QWK assigns larger penalties to predictions that differ by multiple severity grades than to those differing by only one grade. Consequently, it provides a more clinically meaningful evaluation of model performance for ordinal diabetic retinopathy grading.

All classification metrics were computed from the confusion matrix, which consists of true positives (TP), true negatives (TN), false positives (FP), and false negatives (FN). The definitions and mathematical formulations of these evaluation metrics are summarized in Table 3. For all classification metrics, values closer to one indicate better predictive performance, whereas lower loss values indicate better model convergence. The reported overall classification results represent the mean and standard deviation obtained across the five independent runs. To further analyze the learning behavior of each architecture, the weighted cross-entropy loss was employed as the optimization objective during training, while training and validation loss curves were monitored to assess model convergence. The model corresponding to the lowest validation loss was selected as the final model for testing.

In addition to prediction performance, the computational efficiency of each architecture was also investigated. The number of trainable parameters was reported as a measure of model complexity, while inference time was measured to assess the time required to generate predictions for unseen retinal fundus images. These complementary measures provide practical insight into the trade-off between predictive performance and computational cost, which is particularly important when selecting models for real-world diabetic retinopathy screening systems.

## 5. Experimental Results and Comparative Analysis

This section presents the experimental results obtained from the six pretrained deep learning models for five-class diabetic retinopathy grading. The performance of the CNN-based and transformer-based architectures is evaluated using the metrics described in Section 4.3 under the same experimental configuration to ensure a fair comparison. The analysis focuses on three aspects: overall classification performance, learning behavior during training, and computational efficiency. Finally, the strengths and limitations of each architecture are discussed to provide practical insights into selecting appropriate deep learning models for automated DR screening.

### 5.1. Overall Classification Performance

**Figure 3:**
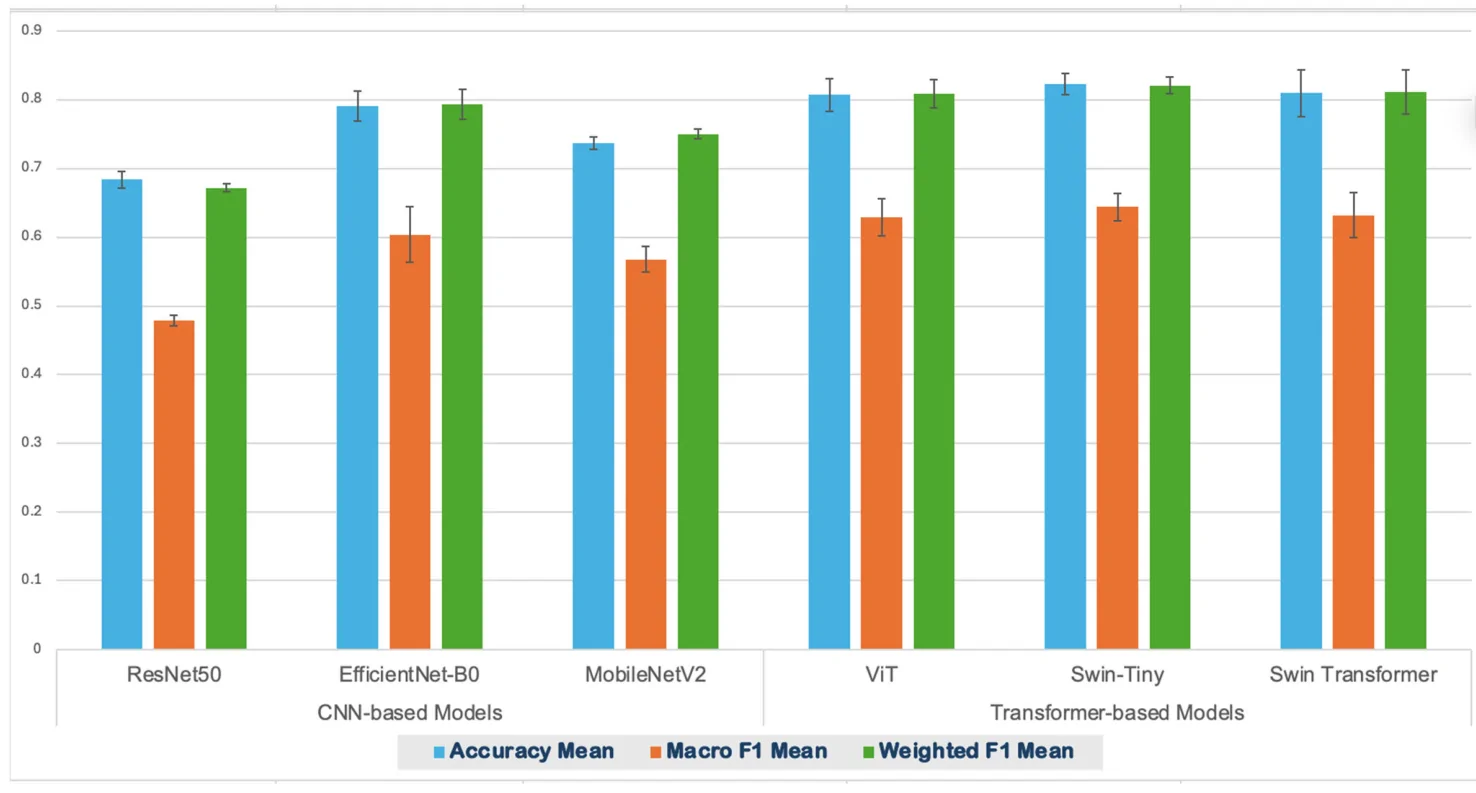
Comparison of the overall classification performance of the evaluated CNN-based and transformer-based models on the independent test set in terms of Accuracy, Macro F1-score, and Weighted F1-score. Error bars indicate variability across five independent runs.

The overall classification performance of the evaluated CNN-based and transformer-based models on the independent test set using accuracy, precision, recall, F1-score, AUC, and QWK is reported in Table 4 and illustrated in Figure 3. To provide a comprehensive evaluation under the imbalanced class distribution of the APTOS 2019 dataset, both macro-average and weighted-average performance metrics are reported. Macro-average metrics assign equal importance to all diabetic retinopathy severity classes, regardless of sample size, making them more suitable for evaluating recognition of minority classes. In contrast, weighted-average metrics account for the class distribution by assigning greater importance to majority classes, thereby reflecting the overall predictive performance on the complete dataset.

Overall, the transformer-based models generally achieved higher mean performance than the CNN-based models across most evaluation metrics. Among the CNN architectures, EfficientNet-B0 achieved the strongest performance, with an accuracy of 0.791, QWK of 0.850, macro F1-score of 0.604, and weighted F1-score of 0.793. Among the evaluated CNN architectures, EfficientNet-B0 achieved the highest mean performance across the main classification metrics, followed by MobileNetV2 and ResNet50. ResNet50 showed the weakest performance, particularly in macro F1-score (0.479), suggesting limited ability to correctly classify minority DR severity grades.

Among the transformer-based models, Swin-Tiny achieved the highest mean accuracy (0.823), QWK (0.898), macro precision (0.667), macro F1-score (0.644), weighted recall (0.823), and weighted F1-score (0.821), resulting in the highest mean values across several of the main classification metrics. The highest mean QWK value further indicates the strongest agreement between the predicted and true DR severity grades while accounting for the ordinal nature of the classification task. Swin Transformer achieved the highest macro recall (0.640), weighted precision (0.832), and weighted AUC (0.966), while obtaining a QWK (0.897) comparable to that of Swin-Tiny. ViT also demonstrated competitive performance, particularly in terms of weighted AUC (0.957) and weighted F1-score (0.809). The relatively small differences between ViT, Swin-Tiny, and Swin Transformer indicate that transformer-based architectures performed comparably, with different architectures achieving the highest mean values across different metrics. The small standard deviation values across the five separate runs indicate generally stable performance across different random seeds. Particularly, QWK showed low variability among the transformer-based models, with standard deviations ranging from 0.010 to 0.015. Although some variation was observed across individual classification metrics, the overall performance patterns were still consistent across the repeated runs.

A noticeable difference is observed between the macro-average and weighted-average metrics across all evaluated models. In general, the weighted-average values were consistently higher than their corresponding macro-average values. This behavior is expected due to the imbalanced distribution of the APTOS 2019 dataset, where the majority classes contribute more heavily to the weighted evaluation metrics. For example, ResNet50 achieved a weighted F1-score of 0.672, whereas its macro F1-score decreased to 0.479, indicating substantially weaker performance on the minority DR severity grades. Similar trends were observed for EfficientNet-B0 and MobileNetV2. In comparison, the transformer-based models generally exhibited smaller gaps between the macro and weighted metrics, suggesting more balanced classification performance across both majority and minority classes.

The AUC and QWK results provide additional insight into model performance. All transformer-based models achieved weighted AUC values above 0.95, with Swin Transformer obtaining the highest mean weighted AUC (0.966), followed closely by Swin-Tiny (0.964). Among the CNN-based models, EfficientNet-B0 also achieved a comparable weighted AUC of 0.953. The transformer-based models obtained higher mean QWK values than the evaluated CNN architectures, with Swin-Tiny and Swin Transformer achieving 0.898 and 0.897, respectively. These results indicate strong discrimination and ordinal agreement among the leading models, while the relatively small differences in AUC further emphasize the comparable performance of the highest-performing architectures.

Finally, to determine whether the observed performance differences were statistically meaningful, statistical analysis was conducted across the five independent runs using QWK, macro F1-score, and weighted F1-score. The Friedman test [42] was first applied to assess overall differences among the six models, followed by pairwise Wilcoxon signed-rank tests with Holm correction [42] to account for multiple comparisons. The Friedman analysis identified significant overall differences among the evaluated architectures for all three metrics (Holm-adjusted *p* < 0.05), confirming that model architecture had a measurable effect on classification performance. Post-hoc analysis showed that the differences among individual leading models were not statistically significant after correction, indicating comparable performance among the strongest architectures despite differences in their mean values. Accordingly, although Swin-Tiny achieved the highest mean QWK, macro F1-score, and weighted F1-score across the five runs, these differences should be interpreted as differences in mean performance rather than statistically significant superiority over the other leading models.

### 5.2. Per-Class Performance Analysis

**Figure 4:**
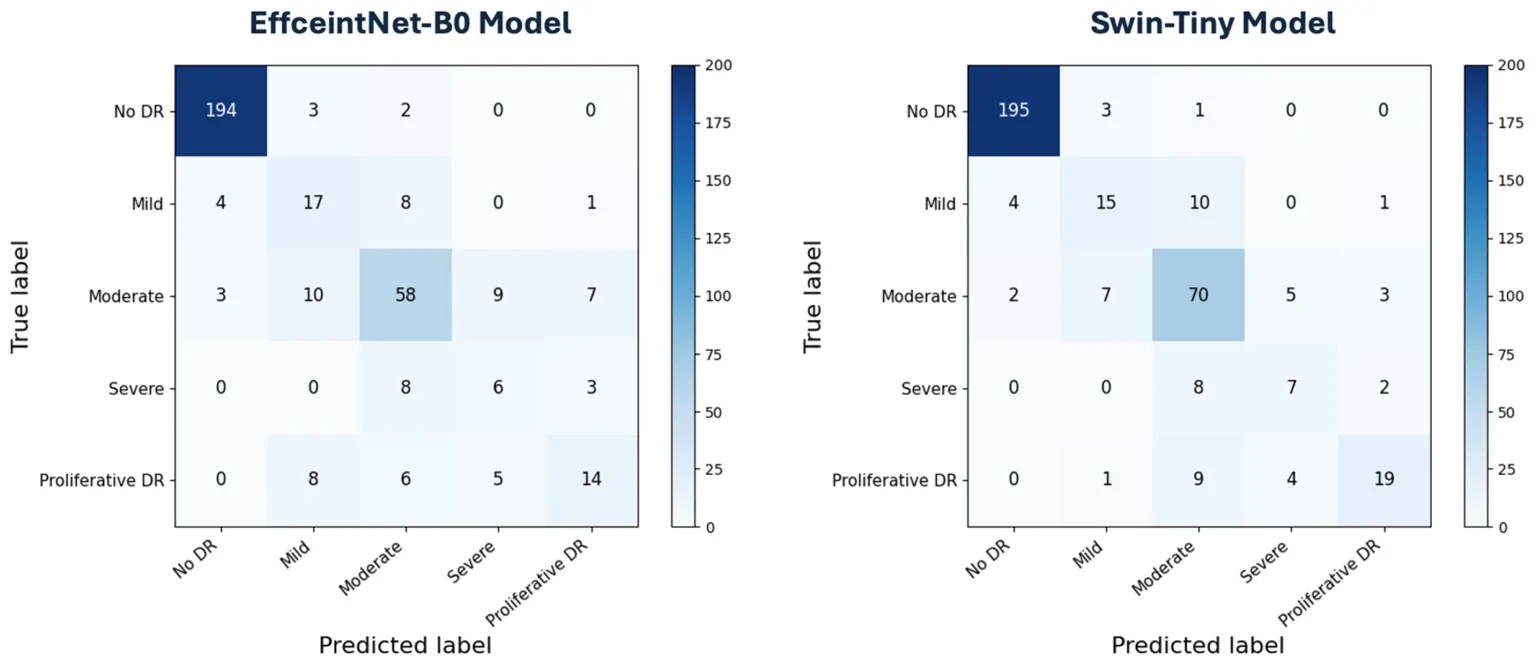
Confusion matrices from a representative run (seed 42) for EfficientNet-B0 and Swin-Tiny on the independent test set for five-class DR grading.

To further examine the classification behavior of the best-performing CNN-based model (EfficientNet-B0) and transformer-based model (Swin-Tiny), a per-class analysis was conducted using precision, recall, and F1-score for each diabetic retinopathy (DR) severity grade. The per-class performance metrics reported in Table 5 correspond to the mean and standard deviation obtained across five independent runs. In addition, to quantify the uncertainty associated with the finite number of test samples, class-specific 95% confidence intervals (CIs) were estimated using 2000 bootstrap resamples of the test predictions for both models. The resulting confidence intervals are reported in Table 6. To provide a qualitative visualization of the classification behavior, the corresponding confusion matrices from a representative test run are presented in Figure 4.

Overall, Swin-Tiny demonstrated better per-class performance than EfficientNet-B0 across nearly all disease severity grades and evaluation metrics. The main exception was Mild DR recall, where EfficientNet-B0 achieved a higher value (0.553 vs. 0.506), while performance for the No DR class was largely comparable between the two models. Both models achieved excellent performance in identifying the majority class “No DR”, with F1-scores exceeding 0.97, indicating reliable discrimination of healthy retinal images. Performance was lower for the Mild and Moderate classes, reflecting the greater difficulty of distinguishing intermediate DR severity levels. For Mild DR, Swin-Tiny achieved higher precision (0.544) and F1-score (0.517), whereas EfficientNet-B0 achieved higher recall (0.553 vs. 0.506), indicating that it correctly identified a larger proportion of actual Mild DR cases and missed fewer cases at this severity level. For Moderate DR, Swin-Tiny showed a clearer advantage, achieving higher precision (0.710), recall (0.793), and F1-score (0.748) than EfficientNet-B0.

The Severe DR class remained the most challenging for both architectures. Swin-Tiny achieved slightly higher precision (0.368), recall (0.400), and F1-score (0.380) than EfficientNet-B0. The variability observed across the independent runs further indicates that performance estimates for this underrepresented class were less stable than those for the No DR class. The bootstrap analysis further quantified the uncertainty associated with the class-specific estimates, with 95% CIs for the Severe DR F1-score of 0.129–0.572 for EfficientNet-B0 and 0.158–0.588 for Swin-Tiny (Table 6), reflecting the limited representation of this class in the test set.

Swin-Tiny also achieved higher mean performance for Proliferative DR, with precision of 0.740 compared with 0.647 for EfficientNet-B0, recall of 0.503 compared with 0.430, and F1-score of 0.598 compared with 0.515. However, the relatively large standard deviations for several Proliferative DR metrics indicate notable variation across the five runs. Overall, the standard deviations across the five independent runs and the bootstrap confidence intervals provide complementary information on the variability and uncertainty of the class-specific performance estimates, particularly for the underrepresented DR severity grades. This finding highlights the influence of class representation on the stability of per-class performance estimates and provides additional context for the observed differences between the two architectures.

The representative confusion matrices (see Figure 4) further illustrate the class-specific classification patterns. A substantial proportion of the classification errors involved neighboring DR severity grades, consistent with the gradual progression of diabetic retinopathy and the visual similarity between adjacent stages. Both models correctly identified the majority of No DR cases. Mild and Severe DR were frequently misclassified as Moderate, with Moderate also accounting for several misclassifications of Proliferative DR. Swin-Tiny correctly classified more Moderate (70 vs. 58) and Proliferative DR cases (19 vs. 14) than EfficientNet-B0 in the representative run, whereas EfficientNet-B0 correctly identified slightly more Mild cases (17 vs. 15). These patterns are generally consistent with the mean per-class results reported across the five independent runs in Table 5.

### 5.3. Learning Behavior and Convergence

**Figure 5:**
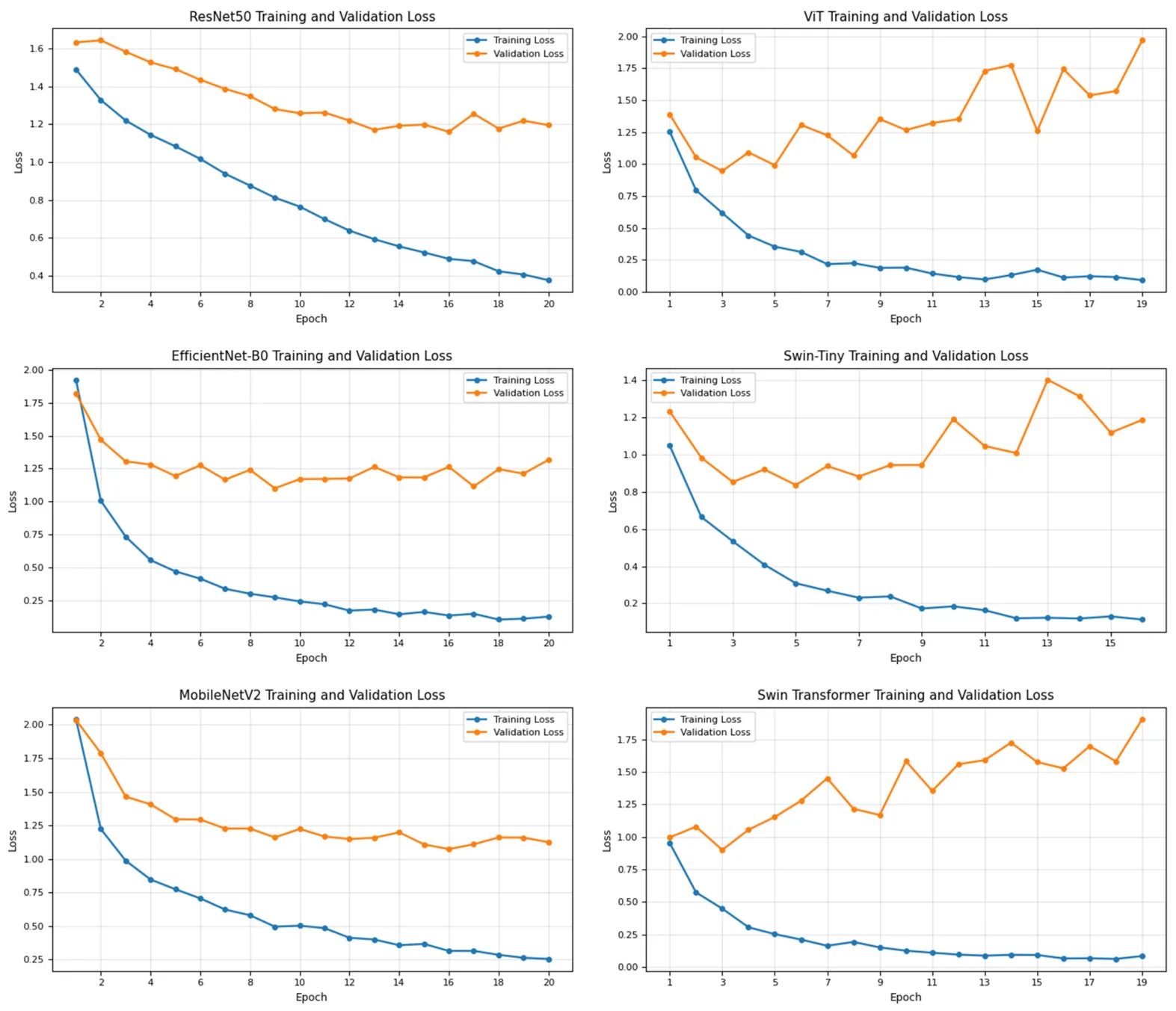
Representative training and validation loss curves from a single experimental run (seed 123) of the evaluated CNN-based and transformer-based models over a maximum of 20 training epochs.

To further analyze the training behavior of the evaluated models, the training and validation loss curves were examined throughout the training process. Figure 5 presents the learning curves from a representative experimental run (seed 123). As shown in Figure 5, all models exhibited a rapid decrease in training loss during the first few epochs, indicating successful optimization. The CNN-based models showed relatively smooth validation curves, whereas the transformer-based models exhibited larger fluctuations in validation loss after the initial training stage, indicating greater sensitivity to continued optimization. As training progressed, the gap between the training and validation losses gradually increased, particularly for the transformer-based models, suggesting increased overfitting during later epochs.

Among the CNN-based models, EfficientNet-B0 and MobileNetV2 showed a relatively rapid reduction in training loss, while ResNet50 exhibited a more gradual convergence pattern. The transformer-based models also converged rapidly, reaching very low training losses within the first few epochs. However, unlike the CNN-based models, their validation losses generally reached their minimum early and subsequently fluctuated or increased despite continued reductions in training loss. This pattern indicates that additional training increasingly optimized the training data without corresponding improvements in validation loss, indicating varying degrees of overfitting across transformer-based architectures.

To further characterize convergence behavior, Table 7 reports the minimum validation loss and its corresponding epoch for the representative run. The transformer-based models achieved their minimum validation losses much earlier than the CNN-based models. Specifically, ViT and Swin Transformer reached their minimum validation losses at epoch 3, while Swin-Tiny reached its minimum at epoch 5. In comparison, ResNet50, EfficientNet-B0, and MobileNetV2 reached their minimum validation losses at epochs 16, 9, and 16, respectively. These results indicate that transformer-based models learn discriminative features rapidly but begin to overfit earlier, while CNN-based models require more training epochs to reach their best generalization performance. The observed differences in convergence behavior also reflect the distinct characteristics of the evaluated CNN- and transformer-based architectures, which may respond in a different way to the common training configuration and reach their optimal validation performance at different stages of training. Early stopping was triggered for ViT, Swin-Tiny, and Swin Transformer at epochs 19, 16, and 19, respectively, whereas all CNN-based models completed the maximum 20 epochs.

### 5.4. Computational Efficiency Analysis

To compare the computational efficiency of the evaluated models, the number of trainable parameters and the inference latency per image were analyzed. Inference latency was measured using a batch size of one under the same hardware environment for all models, following warm-up iterations and repeated measurements. The latency results are reported as mean ± standard deviation across five independent runs, summarized in Table 8.

Among the CNN-based models, MobileNetV2 exhibited the smallest model size, requiring only 2.23 million trainable parameters while achieving the lowest inference latency within the CNN family (7.023 ms/image). EfficientNet-B0 also demonstrated a compact model size, requiring 4.01 M parameters, but exhibited a higher inference latency (9.835 ms/image) than both MobileNetV2 and ResNet50. ResNet50, despite requiring a substantially larger model size (23.52 M parameters), achieved an intermediate inference latency of (7.730 ms/image).

The transformer-based models required considerably more parameters than the CNN models. ViT and Swin Transformer contained approximately 86 M parameters, while Swin-Tiny reduced the model size to 27.52 M parameters. Interestingly, ViT achieved the lowest inference latency among all evaluated models despite having one of the largest model sizes. This observation suggests that inference latency is not solely determined by the number of trainable parameters but is also influenced by the underlying network architecture and its execution efficiency on the target hardware.

Overall, the results demonstrate that model size and inference latency are not directly proportional. While lightweight architectures generally reduce the number of trainable parameters, they do not necessarily achieve the lowest inference latency. These findings highlight the importance of considering predictive performance, model complexity, and inference efficiency when selecting models for automated diabetic retinopathy screening.

### 5.5. Comparison with Related Studies

To provide additional context for the obtained results, the best-performing model in this study was compared with selected studies that also addressed five-class DR grading using the APTOS 2019 dataset. The selected studies employed pretrained or hybrid DL approaches with additional strategies, including Siamese learning [43], patch-based feature extraction [44], and hybrid CNN architectures [45]. The reported accuracy, F1-score, and QWK values are summarized in Table 8, where available. However, several of these studies report a limited set of evaluation metrics, with accuracy frequently serving as the primary performance measure, while F1-score and QWK are not consistently reported. For an imbalanced multi-class dataset such as APTOS 2019, accuracy alone may not fully reflect performance across DR severity grades, particularly the less represented classes. Therefore, Table 9 presents accuracy, F1-score, and QWK for comparison where these measures are available.

As shown in Table 8, the performance of Swin-Tiny is competitive with the selected related studies, although some approaches achieved higher accuracy. These differences may be attributed to the use of additional task-specific strategies, including Siamese learning, patch-based processing, and hybrid architectures, as well as differences in the objectives and experimental designs of the studies. The comparison is also constrained by the limited availability of complementary evaluation metrics in some studies, making it difficult to assess performance beyond overall accuracy. In comparison, the present study considers multiple overall and class-sensitive metrics, including F1-score and QWK, together with per-class performance, computational efficiency, and performance stability across five independent runs. This broader evaluation provides additional insight into model behavior beyond a single aggregate performance measure.

### 5.6. Discussion and Limitations

The experimental results consistently demonstrate that transformer-based architectures outperform CNN-based models for five-class diabetic retinopathy grading. Among the evaluated architectures, Swin-Tiny achieved the highest accuracy, QWK, macro F1-score, and weighted F1-score, while Swin Transformer achieved the highest macro recall, weighted precision, and AUC. ViT also remained competitive across the evaluated metrics. Among the CNN models, EfficientNet-B0 provided the strongest overall performance. The per-class analysis further supported these findings, with Swin-Tiny achieving higher mean performance than EfficientNet-B0 across most DR severity grades and evaluation metrics. However, greater variability was observed for the less represented classes, particularly Severe DR, highlighting the greater difficulty of distinguishing these disease stages. These improvements can be attributed to the self-attention mechanism, which captures both local lesion characteristics and long-range contextual relationships within retinal fundus images.

The computational analysis further demonstrates that model size and inference latency are not directly proportional. MobileNetV2 had the smallest parameter count and the lowest inference latency among the CNN models, while ViT achieved the lowest overall inference latency despite its substantially larger model size. In contrast, Swin Transformer had the largest computational requirements, whereas Swin-Tiny provided a more favorable balance between predictive performance and model complexity, achieving the strongest overall classification performance with substantially fewer parameters than the larger Swin Transformer. These findings suggest that the choice of architecture should depend on the intended deployment scenario. When the primary objective is classification performance, transformer-based models provide a strong choice, with Swin-Tiny achieving the strongest overall performance in this study. However, when model size and computational resources are critical, lightweight CNN models such as MobileNetV2 provide an effective alternative with fewer parameters and competitive classification performance. The inference results further indicate that latency is architecture-dependent and does not necessarily increase with model size, as ViT achieved the lowest inference latency among the evaluated models.

Despite the comprehensive analysis we presented, this study has some limitations. First, the experiments were conducted using a single benchmark dataset, which limits conclusions regarding generalizability across different populations, imaging devices, and acquisition conditions. Second, the imbalanced distribution of APTOS 2019 resulted in relatively few test samples for some DR severity grades, increasing the variability of class-specific performance estimates. Several solutions can be explored for addressing class imbalance and improving the recognition of underrepresented DR severity grades as a future direction. Finally, the study employed a common training configuration across architectures rather than architecture-specific hyperparameter optimization, as the primary objective was to provide a controlled architectural comparison. Finally, the study employed a common training configuration across architectures rather than architecture-specific hyperparameter optimization, as the primary objective was to provide a controlled architectural comparison. Future evaluation across additional retinal datasets and optimized architecture-specific configurations would further establish the generalizability of the observed findings.

## 6. Conclusions

This study presents a comparative analysis of six pretrained CNN- and transformer-based models for five-class diabetic retinopathy grading using retinal fundus images. All models were evaluated under the same preprocessing, training, and evaluation settings to ensure a fair comparison. The results demonstrated that transformer-based architectures consistently outperformed CNN-based models in overall classification performance. Among the evaluated models, Swin-Tiny achieved the strongest overall classification performance, while EfficientNet-B0 provided the strongest CNN baseline. This study highlighted the relationship between predictive performance and computational efficiency. This demonstrates that model size and inference latency are not necessarily proportional, and that architecture selection should consider both predictive and computational requirements.

The comprehensive evaluation across overall and per-class metrics highlights the challenges of distinguishing underrepresented DR severity grades, particularly Severe DR. Future work will extend the evaluation to additional retinal datasets to assess the generalizability of these findings and investigate more recent architectures and architecture-specific optimization. Data augmentation and advanced imbalance-aware learning strategies could also be explored to improve the recognition of underrepresented DR severity grades. Addressing these aspects can further improve and support the development of reliable automated diabetic retinopathy screening systems.

## Figures and Tables

**Table 1:** Distribution of retinal fundus images across the five diabetic retinopathy severity grades and the predefined training, validation, and testing subsets of the APTOS 2019 dataset.

Class	Train	Validation	Test	Total Samples of Each Class
**0—No DR**	1434	172	199	**1805**
**1—Mild DR**	300	40	30	**370**
**2—Moderate DR**	808	104	87	**999**
**3—Severe DR**	154	22	17	**193**
**4—Proliferative DR**	234	28	33	**295**
**Total samples in each split**	**2930**	**366**	**366**	**3662**

Bold indicates the best-performing value for each metric.

**Table 2:** Hyperparameter configuration adopted during model training and fine-tuning for all evaluated architectures.

Hyperparameter	Value
Input image size	224 × 224
Optimizer	AdamW
Learning rate	1 × 10^−4^
Batch size	32
Number of Epochs	20 with patience = 6
Loss function	Weighted Cross Entropy
Sampling strategy	WeightedRandomSampler

**Table 3:** Definitions and mathematical formulations of the prediction evaluation metrics used in this study.

Metrics	Definition	Mathematical Formula
**Accuracy**	Represents the proportion of correctly classified samples among all evaluated images.	*Accuracy* *= (TP + TN)/(TP + TN + FP + FN)*
**Precision**	Measures the reliability of positive predictions by indicating how many samples predicted as positive are actually positive.	*Precision = TP/(TP + FP)*
**Recall ** ** (Sensitivity)**	Measures how effectively the model identifies positive samples from all samples belonging to the positive class. It is also called true positive rate (TPR).	*Recall = TP/(TP + FN)*
**F1-score**	Harmonic mean of precision and recall, providing a balanced evaluation of both metrics.	F1=2×(Precision×Recall)/(Precision+Recall)
**AUC-ROC**	Measures the model’s ability to discriminate between classes across different classification thresholds using the true positive rate (TPR) and false positive rate (FPR).	$AUC={\int_{0}}^{1}\;TPR(FPR)\;d(FPR)$
**QWK**	Measures the agreement between the predicted and actual DR severity grades while accounting for the ordinal relationship between classes using the observed agreement matrix (O), the expected agreement matrix (E), and the quadratic weight matrix (W).	QWK=1−Σ(W ⊙ O)/Σ(W ⊙ E)}

**Table 4:** Overall classification performance (mean ± standard deviation across five independent runs) of the evaluated CNN-based and transformer-based models on the independent test set. The best mean value for each metric is highlighted in bold.

Model	Accuracy	QWK	Macro Average				Weighted Average			
			Precision	Recall	F1 Score	AUC	Precision	Recall	F1 Score	AUC
ResNet50	0.684 ± 0.012	0.801 ± 0.013	0.546 ± 0.013	0.578 ± 0.016	0.479 ± 0.008	0.878 ± 0.007	0.804 ± 0.017	0.684 ± 0.011	0.672 ± 0.006	0.927 ± 0.006
EfficientNet-B0	0.791 ± 0.022	0.850 ± 0.032	0.616 ± 0.038	0.605 ± 0.039	0.604 ± 0.040	0.915 ± 0.014	0.800 ± 0.021	0.791 ± 0.022	0.793 ± 0.022	0.953 ± 0.007
MobileNetV2	0.737 ± 0.009	0.817 ± 0.015	0.565 ± 0.015	0.601 ± 0.019	0.568 ± 0.019	0.904 ± 0.009	0.779 ± 0.006	0.737 ± 0.009	0.750 ± 0.007	0.941 ± 0.004
ViT	0.807 ± 0.024	0.876 ± 0.010	0.648 ± 0.041	0.635 ± 0.015	0.629 ± 0.027	0.918 ± 0.010	0.825 ± 0.017	0.807 ± 0.024	0.809 ± 0.021	0.957 ± 0.003
Swin-Tiny	**0.823** ± 0.015	**0.898** ± 0.010	**0.667** ± 0.028	0.636 ± 0.013	**0.644** ± 0.020	0.931 ± 0.008	0.826 ± 0.010	**0.823** ± 0.015	**0.821** ± 0.012	0.964 ± 0.005
Swin Transformer	0.810 ± 0.034	0.897 ± 0.015	0.658 ± 0.035	**0.640** ± 0.020	0.632 ± 0.033	**0.935** ± 0.013	**0.832** ± 0.008	0.810 ± 0.034	0.811 ± 0.032	**0.966** ± 0.004

**Table 5:** Per-class classification performance (mean ± standard deviation across five independent runs) of the best-performing CNN-based model (EfficientNet-B0) and transformer-based model (Swin-Tiny) on the independent test set. **Bold** indicates the best mean value for each metric within each DR severity grade.

DR Severity Grade	EfficientNet-B0			Swin-Tiny		
	Precision	Recall	F1-Score	Precision	Recall	F1-Score
No DR	0.978 ± 0.009	0.969 ± 0.004	0.973 ± 0.002	**0.974** ± 0.010	**0.973** ± 0.002	**0.973** ± 0.005
Mild	0.449 ± 0.073	**0.553** ± 0.004	0.494 ± 0.058	**0.544** ± 0.103	0.506 ± 0.125	**0.517** ± 0.031
Moderate	0.660 ± 0.043	0.682 ± 0.048	0.671 ± 0.041	**0.710** ± 0.032	**0.793** ± 0.202	**0.748** ± 0.122
Severe	0.344 ± 0.067	0.388 ± 0.089	0.364 ± 0.072	**0.368** ± 0.076	**0.400** ± 0.101	**0.380** ± 0.037
Proliferative	0.647 ± 0.133	0.430 ± 0.091	0.515 ± 0.10	**0.740** ± 0.076	**0.503** ± 0.107	**0.598** ± 0.074

**Table 6:** Class-specific 95% confidence intervals (CI) (lower-upper) for EfficientNet-B0 and Swin-Tiny estimated using 2000 bootstrap resamples of the test predictions.

DR Severity Grade	EfficientNet-B0			Swin-Tiny		
	Precision 95% CI	Recall 95% CI	F1-Score 95% CI	Precision 95% CI	Recall 95% CI	F1-Score 95% CI
No DR	0.951–1.000	0.940–0.990	0.956–0.988	0.947–1.000	0.945–0.995	0.957–0.987
Mild	0.275–0.633	0.367–0.733	0.320–0.647	0.387–0.741	0.267–0.767	0.340–0.667
Moderate	0.547–0.756	0.552–0.793	0.556–0.754	0.632–0.787	0.655–0.943	0.652–0.825
Severe	0.130–0.571	0.118–0.706	0.129–0.572	0.158–0.636	0.176–0.647	0.158–0.588
Proliferative	0.393–0.909	0.212–0.667	0.280–0.741	0.556–0.920	0.303–0.697	0.417–0.758

**Table 7:** Best validation loss, corresponding training epoch, and early stopping information for the evaluated DL models in the representative run (seed 123).

Model	Best Validation Loss	Epoch of Best Model	Early Stopping
ResNet50	1.1598	16	No
EfficientNet-B0	1.1008	9	No
MobileNetV2	1.0728	16	No
ViT	0.9462	3	Yes (epoch 19)
Swin-Tiny	0.8369	5	Yes (epoch 16)
Swin Transformer	0.9000	3	Yes (epoch 19)

**Table 8:** Model complexity and inference efficiency of the evaluated CNN-based and transformer-based architectures. Inference latency is reported as mean ± standard deviation across five independent runs.

Model	Number of Parameters	Mean Inference Latency (ms/Image)
ResNet50	23,518,277	7.730 ± 0.083
EfficientNet-B0	4,013,953	9.835 ± 0.171
MobileNet	2,230,277	7.023 ± 0.181
ViT	85,802,501	6.225 ± 0.012
Swin Tiny	27,523,199	12.341 ± 0.175
Swin Transformer	86,748,349	21.647 ± 0.065

**Table 9:** Comparison of the proposed approach with selected related studies for five-class diabetic retinopathy grading using the APTOS 2019 dataset. NR: not reported.

Study	Model/Method	Accuracy	F1 Score	QWK
Tariq et al. (2024) [43]	Siamese + VGG16	0.810	NR	0.890
	Siamese + ResNet50	0.790	NR	0.860
Kobat et al. (2022) [44]	Patch Division-Based + DenseNET	0.859	0.721	NR
Yasashvini et al. (2022) [45]	CNN	0.756	NR	NR
	CNN + DenseNet	**0.962**	NR	NR
Best Model in this study	Swin-Tiny	0.823	**0.821**	**0.898**

Bold indicates the best-performing value for each metric.

## Data Availability

The APTOS 2019 dataset used in this study was downloaded from the MariaHerreroT Kaggle repository and is publicly available at: (https://www.kaggle.com/datasets/mariaherrerot/aptos2019 (accessed on 20 May 2026)). The dataset can also be accessed programmatically using the kagglehub Python package. The implementation code used in this study is publicly available in the corresponding GitHub repository: https://github.com/MahaThafar/Diabetic-Retinopathy-CNN-Transformer-Comparison/tree/main (accessed on 26 August 2026).

## Abbreviations

The following abbreviations are used in this manuscript:

AI	Artificial Intelligence
APTOS	Asia Pacific Tele-Ophthalmology Society
AUC	Area Under the Receiver Operating Characteristic Curve
CLAHE	Contrast Limited Adaptive Histogram Equalization
CNN	Convolutional Neural Network
DR	Diabetic Retinopathy
DL	Deep Learning
FP	False Positive
FN	False Negative
ICDRSS	International Clinical Diabetic Retinopathy Disease Severity Scale
QWK	Quadratic Weighted Kappa
ROC	Receiver Operating Characteristic
ReLU	Rectified Linear Unit
RGB	Red, Green, and Blue
TP	True Positive
TN	True Negative
TL	Transfer learning
ViT	Vision Transformer
